# Prevalence and Associated Risk Factors of Anemia Among Hospitalized Children in a Tertiary Level Hospital in Botswana

**DOI:** 10.1177/2333794X231156059

**Published:** 2023-02-20

**Authors:** Aobakwe Betty Siamisang, Alemayehu M. Gezmu, Jeremy S. Slone, Lesego Gabaitiri, Thuso David, Bathusi Phetogo, Dipesalema Joel

**Affiliations:** 1 University of Botswana, Gaborone, Botswana; 2 St Jude Children’s Research Hospital, Memphis, TN, USA

**Keywords:** anemia, iron deficiency, risk factors for anemia, pediatrics, hemoglobin

## Abstract

Anemia is a global health concern and has been associated with long term cognitive and behavioral adverse effects. A cross sectional study was conducted to determine the prevalence of and risk factors for anemia in infants and children between 6 months to 5 years of age admitted to a tertiary hospital in Botswana. Baseline full blood count of every patient admitted during the study period was assessed to determine if anemia was present. Data were collected from patient’s medical inpatient chart, electronic medical record (Integrated Patient Management System (IPMS)), and through interviewing parents and caregivers. Multivariate logistic regression model was used to identify risk factors of anemia. A total of 250 patients were included in the study. Prevalence of anemia in this cohort was 42.8%. There were 145 (58%) males. Of the patients with anemia, 56.1%, 39.2%, and 4.7% had mild, moderate, and severe anemia, respectively. Microcytic anemia consistent with iron deficiency was identified in 61 (57%) patients. Age was the only independent predictor of anemia. Children aged 24 months and more had a 50% lower risk of having anemia than their younger counterparts (odds ratio (OR) 0.52; 95% Confidence Interval (95% CI) 0.30 to 0.89). The findings of this study demonstrate anemia as a serious health concern in the pediatric population in Botswana.

## Introduction

Anemia is defined as hemoglobin below the fifth percentile for age. In children 6 months to 5 years of age this translates to hemoglobin level less than 11 g/dl.^1^ Severity of anemia is classified as Mild (10-10.9 g/dl), moderate (7-9.9 g/dl), and severe (<7 g/dl) based on hemoglobin concentration.^1^ The mechanisms that result in anemia are numerous. Many of the causes result in anemia through increased red cell destruction, impaired red blood cell production and acute or chronic blood loss.^2^ Some of the implicated causes of anemia include infections such as malaria, hookworm, and HIV; and medications such as antiretroviral, anti-tuberculous, chemotherapeutic agents and some antibiotics. Additionally, inflammatory diseases, chronic kidney disease and malignancies are known causes of anemia of chronic illness. Furthermore, micronutrient deficiencies of iron, vitamin B12 and folic acid are important causes of anemia.^3,4^ Of micronutrient deficiencies, iron deficiency is the most common cause in children.^5,6^

Serum ferritin level is the gold standard for investigating body iron stores in children.^6,7^ However, in low socio-economic settings, it is not always possible to do iron studies. In Botswana, iron studies are outsourced to private health facilities resulting in additional costs to the Botswana Government or the family. However, red cell distribution width (RDW) has proven sensitivity and specificity in the diagnosis of iron deficiency anemia (IDA).^8,9^ In a study of 2091 children aged 1 to 3 years in India, a combination of hemoglobin concentration of less than or equal to 10 g/dl and RDW of more than 15% was 90% specific and 99% sensitive for IDA.^9^ In a study of patients aged 5 months to 50 years, RDW was found to be much higher in IDA (36.2%-55.2%) than in thalassemia trait (14.7%-24.9%).^10^

Anemia is a global health problem.^3,11^ Globally, anemia affects 1.62 billion people.^3^ The highest prevalence is in preschool children aged 6 to 59 months with the highest proportion seen in Africa.^3^ The prevalence of anemia in Africa has been shown to be high.^12,13^ In Cape Verde, a study identified a prevalence of 51.8% in 2014.^12^ Additionally, a much higher prevalence was demonstrated in Mali and Benin where anemia in pre-school children was found to be 83% and 82%, respectively.^14^ A hospital based study done in preschool children in a Nigerian outpatient department also showed anemia at prevalence of 49.7%.^15^ Stunting, malaria positivity and recent illness were associated with lower hemoglobin levels in a survey of children in 3 African countries; Malawi, Tanzania, and Ghana.^16^

The World Health Organization global estimates for anemia from 1993 to 2005, described prevalence of anemia in Botswana as a moderate public health concern at 38% in preschool children.^3^ However, in 2015, the WHO released another report of global estimates of anemia. In this report Botswana had anemia prevalence in preschool children estimated at 43%, which is of severe public health significance.^4^

Anemia remains a major contributor to under 5 mortality rates in sub-Saharan Africa.^17^ Chronic anemia is associated with significant morbidity including loss of productivity from impaired work capacity, cognitive impairment and increased susceptibility to infections.^18^ This is particularly true for iron deficiency anemia.^2,5,19^ The maximum effects of cognitive impairment have been shown in the ages of 6 to 18 months when iron demand is increased due to rapid growth.^20,21^ A study of 40 children aged 6 months to 12 years with IDA in Egypt showed a significant reduction on humoral immunity (Immunoglobulin G levels), interleukin 6 levels and non-specific immunity (phagocytic activity and oxidative bursts) compared with age-matched controls.^22^ Another case control study in India showed that IDA caused impairment in cell-mediated immunity that improved after 3 months of iron supplementation.^23^ This study aimed to determine prevalence of anemia and associated risk factors in hospitalized children in Botswana.

## Methods and Materials

A cross-sectional study was conducted at a tertiary level hospital in Gaborone, Botswana from November 2017 to May 2018. This 560-bed tertiary level hospital caters for patients mainly from southern part of the country. Pediatric medical and surgical wards, where study participants were identified, have a capacity of 70 beds and admit 200 patients per month. At admission, patients typically routine laboratory tests such as full blood count. The hospital uses Integrated Patients Management System (IMPS) as an electronic medical record.

Infants and children between age 6 months to 5 years, admitted during the study period, and had a full blood count test result were included in the study. Patients who received a blood transfusion in the last 3 months prior to admission, had acute blood loss, or were on treatment for cancer were excluded.

The primary investigator and a study assistant collected clinical and sociodemographic data from patients’ parents/caregivers and medical record files. A sample size of 250 participants was calculated assuming the prevalence of anemia in Botswana at 43%.^4^

Anemia was defined as hemoglobin (Hb) below the fifth percentile for age. According to WHO,^1^ in a child aged 6 months to 5 years, this is hemoglobin less than 11 g/dl. Whereas severity of anemia was classified as mild, moderate, and severe when Hb levels were between 10 and 10.9 g/dl, 7 and 9.9 g/dl and less than 7 g/dl, respectively. Furthermore, Hb concentration of less than or equal to 10 g/dl and RDW of more than 15%,^8,9^ was used as proxy indicators of presumed iron deficiency as serum ferritin level measurement was not routinely available in the hospital.

Data were analyzed using SPSS version 25 (IBM, Chicago, USA). Prevalence of anemia was calculated as a proportion. Categorical variables were presented as frequency and percentage. Median and interquartile range were used to report continuous variables. Chi-square test was performed to determine group difference for categorical variables. A multivariate logistic regression analysis was used to identify factors associated with anemia. Statistical significance was defined at *P*-value of less than 0.05.

## Results

A total of 269 patients were identified for inclusion in the study. Nineteen of the participants were excluded due to missing information. The median age of the participants was 20 months (inter-quartile ranges (IQR) 10, 29 months). There were 145 (58%) males. The majority (98.4%) of the study participants were HIV negative. Infectious diseases were the commonest (56%) cause of admission. None of the study participants had confirmed malaria. The majority (78.8%) of the patients had no chronic illnesses (Table 1).

**Table 1.** Baseline Demographic and Clinical Characteristics of Study Participants.

Variables	Total n (%)	Anemia n (%)	Normal Hb n (%)
Age category			
6-23 months	157 (62.8)	77 (49)	80 (51)
24-60 months	93 (37.2)	30 (32)	63 (68)
Sex			
Male	145 (58)	63 (43)	82 (57)
Female	105 (42)	44 (42)	61 (58)
HIV status			
Positive	4 (1.6)	3 (75)	1 (25)
Negative	246 (98.4)	104 (42)	142 (58)
Patient nutritional status			
Malnutrition	40 (16)	23 (57.5)	17 (42.5)
Normal	210 (84)	84 (40)	126 (60)
Birth weight			
Low birth weight	39 (15.6)	18 (46.2)	21 (53.8)
Normal birth weight	211 (84.4)	89 (42.2)	122 (57.8)
Feeding in first 6 month			
Exclusive breast milk feeding	134 (53.6)	50 (37)	84 (63)
Exclusive formula	53 (21.2)	28 (52.8)	25 (47.2)
Long life cow’s milk	1 (0.4)	1 (100)	0 (0)
Mixed feeding	62 (24.8)	28 (45.2)	34 (54.8)
Admission diagnosis			
Infectious disease	141 (56.4)	63 (44.7)	78 (55.3)
Paraffin ingestion	25 (10)	9 (42.8)	16 (57.1)
Febrile convulsions	21 (8.4)	4 (19)	17 (81)
Hematology/oncology diagnosis	5 (2)	2 (40)	3 (60)
Others	58 (23.2)	28 (48.3)	30 (51.7)
Chronic illnesses			
No	197 (78.8)	87 (44.2)	110 (55.8)
Yes	53 (21.2)	20 (37.7)	33 (62.3)

The prevalence of anemia among the study participants was 42.8%. The median hemoglobin level was 11.3 g/dl (IQR 10.3, 12.1). Of the 107 patients with anemia, 56.1%, 39.2%, and 4.7% had mild, moderate and severe anemia, respectively. IDA, based on low Hb, low MCV and high RDW, was found in 61 (57%) patients with anemia and 61 (24.4%) of all study participants.

Age greater than 24 months was associated with 50% decreased risk for anemia on a multivariate analysis after accounting for gender, HIV, nutrition status and birth weight (adjusted Odds Ratio (aOR) 0.518, 95% Confidence Interval 0.302 to 0.889, *P* = .017) (Table 2).

**Table 2.** Uni and Multivariate Logistic Regression Analysis for Risk Factors of Anemia in the Study cohort.

	Univariate		Multivariate	
Variables	Crude odds ratio (95% CI)	*P*-value	Adjusted odds ratio (95% CI)	*P*-value
Age				
<24 months	1		1	
≥24-59 months	0.495 (0.290-0.845)	.010	0.518 (0.302-0.889)	.017
Sex				
Male	0.939 (0.565-1.560)	.808	0.890 (0.514-1.541)	.677
Female	1		1	
HIV status				
Negative	1		1	
Positive	4.096 (0.420-39.937)	.225	5.362 (0.416-69.112)	.198
WAZ				
Underweight	1		1	
Normal weight	0.692 (0.375-1.278)	.240	0.627 (0.246-1.601)	.407
HAZ				
Stunted	1		1	
Normal height	0.929 (0.502-1.719)	.815	0.937 (0.506-1.735)	.837
WFH				
<−2 z-score	2.029 (1.023-4.026)	.043	1.873 (0.935-3.750)	.076
>−2 z-score	1		1	
Birth weight				
<2500 g	1		1	
≥2500 g	0.851 (0.428-1.691)	.645	0.966 (0.454-2.054)	.882

Abbreviations: HIV, human immunodeficiency virus; WAZ, weight for age z-score; HAZ, height for age z-score; WFH, weight for height z-score.

## Discussion

In this cohort of hospitalized children under the age of 5 in Botswana, the prevalence of anemia was 43%. The majority of patients were found to have mild anemia. Iron deficiency anemia (IDA) was presumed to be the cause in 57% of the anemic patients. Age was the only independent predictor of anemia in this study cohort with children more than 24 months of age having a 50% lower risk of having anemia compared to younger children.

The study findings on prevalence of anemia in Botswana was similar to a WHO report released in 2015 which was estimated to be 43%.^4^ Furthermore, the prevalence of anemia from this study is also similar to other studies conducted in LMIC’s.^24-26^ Anaemia prevalence in preschool children were 38.8%, 51.2%, and 52% in Haiti, Brazil, and South Africa, respectively. However, this study’s estimate was lower than what was identified in Ghana, Burma, and Uganda where the prevalence was at 73.1%, 72.6%, and 58.8%, respectively.^13,27,28^ In these countries, malaria was a common diagnoses in the studied participants and could therefore explain the markedly high prevalence of anemia. None of the study participants in this study’s cohort had confirmed malaria. Additionally, a 5 years national passive malaria surveillance in Botswana (2008-2012) showed a 98% reduction in malaria prevalence.^29^ The prevalence of IDA amongst the anemic patients of 57% is consistent with previously published data that indicates IDA is present in more than 50% of anemic patients globally.^6,7,30,31^

In this study, young age was the only significant independent risk factor associated with anemia. Children aged less than 24 months were twice as likely to have anemia at admission compared to children aged more than 24 months. This finding has been demonstrated in other studies conducted in LMIC’s.^12,13,32,33^ In Cape Verde, anemia prevalence was 72% in children aged less than 2 years and 52.2% amongst older children.^12^ Younger children were found to be more than 3 times at risk of having anemia. Similar findings were found in Brazil, Uganda, and Eastern Cuba.^16,24,34^ The increased risk of anemia with younger age could be due to poor iron intake during weaning. Safe complementary feeding in children from 6 months is not always practiced, where the feeding of unmodified cow’s milk in children less than 12 months of age is common in some Sub-Saharan Africa countries, including Botswana.^35-37^

The patients with malnutrition were found to be twice at risk for anemia than those with normal nutrition. While significant in the univariate analysis, this was not maintained in the multivariate analysis. This was in contrast to a Ghanaian study where malnutrition was significantly associated with anemia.^38^

This study has several limitations. Firstly, this is a hospital-based study conducted in acutely ill patients and may not reflect what is happening in the community. Secondly, the lower number of patients from the pediatrics surgical ward (PSW) for whom FBC was not done can lead to selection bias. Thirdly, iron laboratory studies were not conducted for diagnosis of iron deficiency anemia due to lack of resources. Finally, the cross-sectional study design is limited in its ability to detect direct cause-effect relation between independent variables and the outcome variables. Further studies are therefore needed in communities, child welfare clinics and preschools to further quantify the magnitude of anemia in Botswana.

## Conclusion

The findings of this study demonstrate anemia as a serious health concern in young children win Botswana which requires improved screening practices and prompt intervention. Further studies in preschool children are required in the community to assess the magnitude of anemia in Botswana children.
